# SmartBuildSim: An Open-Source Synthetic-Twin Framework for Reproducible AI Benchmarking in Smart-Building Analytics

**DOI:** 10.3390/s25237263

**Published:** 2025-11-28

**Authors:** Tymoteusz Miller, Irmina Durlik, Agnieszka Nowy, Ewelina Kostecka

**Affiliations:** 1 Institute of Marine and Environmental Sciences, University of Szczecin, 70-383 Szczecin, Poland; 2 Faculty of Data Science and Information, INTI International University, Nilai 71800, Malaysia; 3 Faculty of Navigation, Maritime University of Szczecin, 70-500 Szczecin, Poland; 4 Faculty of Mechatronics and Electrical Engineering, Maritime University of Szczecin, 70-500 Szczecin, Poland

**Keywords:** smart building, synthetic data, AI benchmarking, forecasting, anomaly detection, reinforcement learning

## Abstract

**Highlights:**

**What are the main findings?**

- SmartBuildSim provides a lightweight synthetic-twin framework that generates reproducible smart-building sensor streams with configurable trend, seasonality, noise, drift, and missingness.
- The framework integrates ready-to-use AI pipelines for forecasting, anomaly detection, clustering, and reinforcement learning, enabling reproducible benchmarking across diverse building scenarios.

**What are the implications of the main findings?**

- Researchers and practitioners can use SmartBuildSim as a transparent, extensible testbed to evaluate AI methods before applying them to high-fidelity simulators or real sensor networks.
- The tool bridges the gap between limited real-world sensor datasets and complex physical simulations, fostering reproducibility and accelerating innovation in smart sensing and digital twin applications.

**Abstract:**

This paper introduces SmartBuildSim, an open-source synthetic-twin framework that generates configurable and reproducible multi-sensor building streams using lightweight statistical models with tunable trend, seasonality, correlation, delays, and anomaly mechanisms. Deterministic seeding ensures experiment-level reproducibility, while modular pipelines support unified evaluation across forecasting, anomaly detection, and RL tasks. A comprehensive validation against an ASHRAE Great Energy Predictor III reference signal demonstrates that the synthetic data capture realistic magnitude and variability (KS ≈ 0.32; DTW ≈ 9.69), while preserving interpretable and controllable temporal structure. Benchmark results show that simple linear models achieve strong forecasting performance (RMSE ≈ 21.27), IsolationForest reliably outperforms LOF in anomaly detection (F1 ≈ 0.17 vs. 0.10), and Soft-Q Learning provides substantially more stable RL convergence than tabular Q-learning (variance reduced by >95%). Scenario-level analyses further illustrate reproducible daily cycles, zone-specific differences, and the scalability of model behaviour across building configurations. By combining declarative YAML configurations, deterministic randomness management, and an extensible scenario engine, SmartBuildSim provides a transparent and lightweight alternative to high-fidelity building simulators. The framework offers a practical, reproducible testbed for smart-building AI research, bridging the gap between simplistic synthetic datasets and complex physical digital twins. All code, tables, figures, and a Google Colab workflow are openly available to ensure full replicability.

## 1. Introduction

The increasing deployment of sensors in modern buildings has opened new opportunities for forecasting energy demand, detecting anomalies in operation, and optimizing control strategies through artificial intelligence [1,2,3]. Artificial intelligence (AI) technologies in smart buildings can reduce energy consumption through improved control, reliability, and automation [2]. AI-based methodologies are crucial for identifying inefficiencies, forecasting future energy requirements, and mitigating energy wastage [3]. AI also enables buildings to participate in energy markets by predicting real-time supply and demand and plays a significant role in fault detection and diagnostics [3].

However, research in this area is still limited by the scarcity of open datasets and the difficulty of establishing reproducible benchmarks [4]. Critical research gaps include the lack of large-scale empirical validation and challenges in AI scalability [4]. Real sensor data are often proprietary, noisy, or incomplete, which restricts their usefulness for systematic evaluation of algorithms [5]. Data quality issues are a significant factor influencing the adoption of data-driven technologies in smart buildings [5]. Moreover, differences in measurement granularity, sensor placement, and missing values make it challenging to compare methods across studies [5].

Synthetic data generation provides a viable alternative for overcoming these barriers [6,7,8,9]. By producing controlled, reproducible streams that mimic the statistical properties of building sensors, synthetic frameworks allow researchers to test algorithms under known conditions and share results that can be replicated by others [10]. The ability to introduce controlled imperfections—such as noise, drift, or missing values—further strengthens their role as benchmarking tools [11].

In this context, we present SmartBuildSim, an open-source framework for generating synthetic smart-building sensor data and providing AI-ready pipelines for forecasting, anomaly detection, clustering, and reinforcement learning. Unlike traditional building simulators that focus on high-fidelity physical models, SmartBuildSim generates time-series data using configurable components such as trend, seasonality, and stochastic noise. The framework also includes mechanisms to inject realistic imperfections, including systematic drift and structured missingness, which are commonly observed in real sensor logs.

The library provides both a Python (3.10 version) API and a command-line interface (CLI) [12,13,14,15,16,17,18]. This dual approach allows for interactive scripting and integration into high-performance computing workflows [13,18]. The use of a CLI can enhance reproducibility and comparison of research results [14,17,19].

The provided sources do not contain information about the library being built on Typer, using typed Pydantic configurations for schema validation, or guaranteeing reproducibility through deterministic seeding.

Reproducibility is guaranteed through deterministic seeding, ensuring that every run produces identical outputs given the same configuration. Two reference scenarios—*office-small* and *campus*—are distributed with the package, allowing one-command replication of the entire workflow.

SmartBuildSim therefore fills an important gap between high-fidelity building simulation tools and purely data-driven AI studies. By emphasizing reproducibility, transparency, and AI readiness, it enables researchers to benchmark algorithms in a controlled yet realistic setting, and to advance the state of the art in smart-building analytics. SmartBuildSim is intentionally designed as a low-fidelity but high-reproducibility synthetic-twin framework. In contrast to high-fidelity simulators such as EnergyPlus or TRNSYS, the goal is not to replicate full thermodynamic or occupancy-driven behavior, but to provide transparent, deterministic, and AI-ready data streams suitable for benchmarking, algorithmic prototyping, and controlled methodological studies.

## 2. Related Work

Several frameworks have been developed to support research in smart-building analytics, but they differ considerably in their objectives, fidelity, and suitability for benchmarking artificial intelligence methods.

EnergyPlus, maintained by the U.S. Department of Energy, is the most widely used high-fidelity simulation engine for building energy analysis. It provides detailed models of thermal dynamics, HVAC systems, and envelope properties, and is capable of simulating entire buildings at high resolution [20]. While EnergyPlus is indispensable for design and engineering studies, its complexity and computational requirements make it less suitable for rapid benchmarking of machine learning methods. Moreover, it does not natively provide pipelines for forecasting, anomaly detection, or clustering.

BOPTEST (Building Optimization Performance Test framework) was developed to evaluate advanced building controllers in a reproducible manner [21]. It provides standardized Key Performance Indicators (KPIs) and uses Modelica-based emulators to replicate building dynamics. BOPTEST is typically deployed in Docker containers and exposes a RESTful API for controller interaction. Its primary focus is on controller benchmarking and fault detection, rather than the generation of machine learning–ready datasets.

CityLearn is an environment for multi-agent reinforcement learning in urban building energy systems [22]. It is distributed as a Gymnasium-compatible package and has been used in international challenges on demand response and load shifting. CityLearn’s strength lies in its ability to standardize RL environments across multiple agents, but it does not aim to generate synthetic time-series data streams or to support anomaly detection and clustering tasks.

In contrast, SmartBuildSim [23] was specifically designed to fill the gap between high-fidelity simulators and RL testbeds by offering a lightweight synthetic data generator that emphasizes reproducibility and AI readiness. Instead of detailed physical models, SmartBuildSim produces configurable time-series streams based on trend, seasonality, and stochastic noise. It further supports the injection of missing values and long-term drift, two imperfections that are common in real sensor data but rarely captured in benchmark frameworks. Importantly, SmartBuildSim integrates baseline pipelines for forecasting, anomaly detection, clustering, and reinforcement learning, ensuring that the generated data can be immediately used to evaluate algorithms. By combining deterministic seeding, typed configurations, and a simple CLI, it provides a reproducible and transparent environment for testing smart-building AI methods.

Table 1 summarises the main architectural components of SmartBuildSim, including the configuration layer, data generator, feature engineering utilities, evaluation pipelines, and the validation module. The table highlights how each module interacts with the others through typed Pydantic schemas, deterministic seeding, and a unified data interface based on timestamp–sensor–value records.

## 3. Methods: Architecture of SmartBuildSim

**Figure 1:**
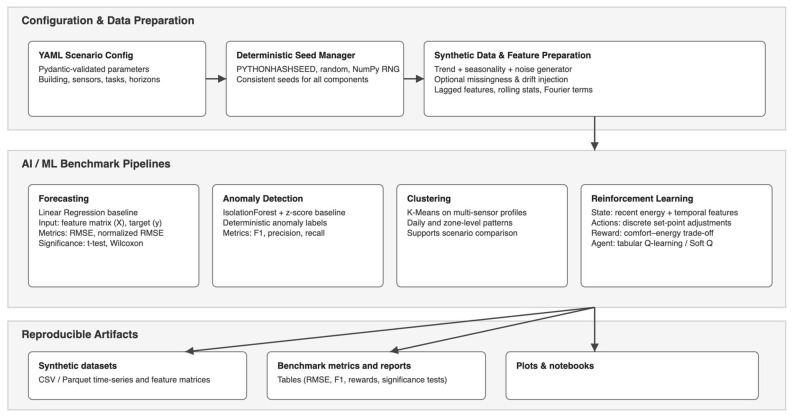
System Architecture of SmartBuildSim.

SmartBuildSim is an open-source, deterministic smart-building simulation toolkit implemented in Python and distributed under the MIT license (Available online: https://github.com/TyMill/SmartBuildSim (accessed on 2 October 2025)). The framework is organised as a modular library with a thin command-line interface (CLI) layer, making it suitable both for interactive experiments in notebooks and for scripted, reproducible benchmark pipelines. Figure 1 illustrates the overall structure, while Table 2 summarizes the specific modules and their capabilities.

At the core of SmartBuildSim lies a lightweight scenario engine that encodes building layouts, zones, and sensor configurations using BIM-style abstractions. Each scenario (e.g., *office-small* or *Innovation Campus*) is defined through a Pydantic-based configuration, which specifies the building structure, the set of sensors to be simulated, and the statistical properties of their signals. These definitions are consumed by the data generator module, which produces multi-sensor time series with configurable trend, seasonality, correlation between streams, time delays, and anomaly injection. The generator operates in a deterministic fashion: a dedicated DeterminismConfig model and helper utilities (configure_determinism, resolve_seed, create_rng) centralise random seed management across NumPy, the Python random module, and library components. As a result, identical configuration files and seeds always yield identical datasets, which is crucial for fair benchmarking and reproducibility [24,25,26,27].

Generated data are represented in a canonical, tidy format with columns such as timestamp, zone, sensor, value, and optional metadata (e.g., anomaly labels). This uniform schema is shared by all subsequent modules [28]. The feature engineering layer converts raw time series into supervised learning matrices by constructing lagged features and prediction horizons aligned with the forecasting and anomaly detection tasks. On top of this, the evaluation subpackage provides unified benchmarks for forecasting, anomaly detection, and reinforcement learning. It wraps standard scikit-learn models (e.g., linear regression, Histogram-based Gradient Boosting, IsolationForest, LOF) and custom RL agents (tabular Q-learning and Soft-Q) into a common interface that handles repeated K-fold evaluation, aggregation of metrics (e.g., RMSE, F1-score, reward), and statistical significance testing [1].

A separate validation module (smartbuildsim.data.validation) enables direct comparison between synthetic and real-world datasets. It accepts any data structured in the same timestamp–sensor–value format and computes distributional statistics, autocorrelations for selected lags, dynamic time warping (DTW) distances, and correlation matrix discrepancies, returning both numeric summaries and qualitative notes. This module is used, for example, to compare the synthetic *office_energy* stream with an ASHRAE Great Energy Predictor III reference meter [29].

All components are orchestrated by a Typer-based CLI that reads declarative YAML configuration files [30]. The user specifies the scenario, data generation settings, model hyperparameters, output paths, and determinism options in a single document, which can be overridden at runtime using dotted keys. The same configuration can drive the full pipeline: BIM initialisation, data generation, model training, anomaly detection, clustering, RL training, and Matplotlib-based visualisations. For interactive use, SmartBuildSim also provides example scripts and Jupyter/Colab notebooks that reproduce all tables and figures reported in the results section using a single, readable workflow.

### 3.1. Data Generation and Configuration

The synthetic data generator [31] is the central component of SmartBuildSim and is responsible for constructing multi-sensor building streams that reflect realistic but controllable temporal dynamics. Each simulation is defined by a declarative Pydantic configuration (DataGeneratorConfig), which specifies the statistical mechanisms governing the behaviour of each sensor. The generator constructs its output by combining several configurable components: baseline trend, diurnal and weekly seasonality, shared and sensor-specific noise, nonlinear dependencies, time delays, and an optional anomaly injection model.

The trend and seasonality components control the deterministic structure of the signal and are expressed through periodic functions and zone-level modifiers [32]. These are complemented by stochastic perturbations, including sensor-level variance, correlated noise shared among related sensors (e.g., temperature–energy links), and optional drift. Nonlinear influences—for example, occupancy affecting CO_2_ concentration or HVAC energy—are implemented through parametric transformations that the user can tune directly in the configuration file.

Time-dependent effects are supported via deterministic and stochastic delays, allowing the generator to model HVAC response lag, ventilation delays, or the time required for occupancy changes to propagate into measurable sensor values. The anomaly model introduces controlled irregularities such as spikes, drops, or regime shifts, with tunable amplitude, duration, and frequency [33]. When enabled, the generator also records a binary anomaly mask, ensuring that all downstream tasks—forecasting, anomaly detection, and RL—have access to the same ground-truth labels.

All generated data adopt the canonical long format used throughout the framework, with each record containing at least a timestamp, zone, sensor, and value. Optional columns such as is_anomaly and value_normalized (created when normalization is enabled) provide additional context for downstream evaluation. Because the generator strictly adheres to the determinism configuration described in Section 3.1, identical seeds and YAML files always produce bitwise-identical datasets, enabling exact replication of all experiments reported in this work.

### 3.2. Validation, Benchmarking, and Learning Workflows

SmartBuildSim includes built-in modules for validating synthetic datasets and benchmarking machine-learning algorithms across forecasting, anomaly detection, clustering, and reinforcement learning tasks. These components share a common interface and operate on the unified data schema described above, ensuring seamless integration across the pipeline.

The validation module (smartbuildsim.data.validation) compares synthetic data streams with real-world reference datasets using a set of distributional, temporal, and correlation-based metrics. Given two datasets with aligned sensor names, the validator computes means, standard deviations, Kolmogorov–Smirnov statistics, lagged autocorrelations, dynamic time warping (DTW) distances, and correlation matrix differences [34]. The output consists of a structured report (JSON-compatible) containing numeric metrics and qualitative notes indicating potential discrepancies. This module is used in Section 4 to compare synthetic office energy readings with a reference subset from the ASHRAE Great Energy Predictor III dataset.

The benchmarking module (smartbuildsim.evaluation.benchmark) provides unified workflows for forecasting, anomaly detection, and reinforcement learning. Forecasting benchmarks rely on automatically constructed lagged feature matrices and evaluate both linear and tree-based models, reporting RMSE across multiple random seeds and K-fold splits. Anomaly detection benchmarks support IsolationForest and LocalOutlierFactor, computing modular F1-scores together with statistical significance tests (paired t-tests and Wilcoxon signed-rank tests) [35]. Reinforcement learning benchmarks evaluate tabular Q-learning and Soft-Q agents implemented in the RL subsystem, comparing their reward distributions and convergence stability across seeds. All benchmarks employ deterministic seed management, ensuring that repeated runs produce identical numerical results.

A separate clustering layer is available for exploratory analysis (e.g., K-Means-based profiling of daily energy cycles), though it is not included in the statistical benchmark suite. All workflows are accessible through both the Python API and a unified Typer-based CLI that executes full YAML-driven pipelines. For transparency and full reproducibility, a Google Colab notebook accompanying this work regenerates all datasets, tables, and figures reported in Section 5.

### 3.3. Scenario Engine and Reference Configurations

SmartBuildSim provides a flexible scenario engine that defines the structural, temporal, and analytical characteristics of each simulated environment. A scenario consists of (i) a building description encoded as a set of zones and sensors, (ii) a data-generation configuration controlling temporal dynamics, noise, delays, and anomalies, and (iii) task-specific configurations for forecasting, anomaly detection, clustering, and reinforcement learning (RL). All scenario components are fully declarative and validated through Pydantic, enabling a transparent and reproducible workflow driven either by YAML files or Python-based specifications.

To demonstrate the reproducibility and extensibility of the framework, two representative scenarios were used in this study: the built-in office-small and a custom Innovation Campus configuration. Both are executed through the same pipeline, ensuring that results from different building layouts remain directly comparable.

The office-small scenario represents a compact two-zone office environment with energy and CO_2_ sensors. It is distributed with the framework and can be instantiated using a single YAML configuration file. An example is shown in Appendix A, where simulation duration, deterministic seed, forecasting horizon, anomaly contamination, clustering sensors, and visualisation targets are specified. This minimal configuration is sufficient to generate a multi-sensor dataset, construct lagged supervised features, run forecasting, anomaly detection, clustering, and RL pipelines, and export all intermediate artefacts in tidy CSV/Parquet formats.

To illustrate extensibility, a more heterogeneous Innovation Campus scenario was constructed manually using the Python API (Appendix B). This configuration describes a four-zone academic building (FabLab, Lecture Hall, Server Room, Green Lab) with a diverse set of temperature, energy, humidity, and CO_2_ sensors. The data-generation settings include 28 simulation days at a 15-min resolution, nonlinear occupancy–HVAC effects, stochastic noise, and moderate seasonal drift. Forecasting, anomaly detection, clustering, and RL tasks are aligned with zone-specific characteristics—for example, forecasting is performed on the stable *serwerownia_energy* series, while anomaly detection focuses on *aula_co2*, where occupancy-driven fluctuations play a stronger role.

Across both scenarios, the experimental workflow remains consistent. The pipeline begins with loading the scenario configuration (YAML or Python) and generating synthetic sensor streams using the deterministic statistical generator. Depending on the configuration, the data may include missing values, drift, anomalies, nonlinear dependencies, shared noise, and sensor-specific delays. For evaluation tasks, the raw time series are transformed into supervised matrices with lagged variables, rolling statistics, Fourier terms, and additional engineered features required by forecasting and anomaly detection models.

Each scenario is then processed using four unified pipelines:

(1) forecasting, using lagged features and RMSE-based evaluation;
(2) anomaly detection, using IsolationForest and LOF on engineered features;
(3) clustering, identifying zone-level behavioural groups through K-Means;
(4) reinforcement learning, using tabular Q-learning or Soft-Q to control thermal or energy-related targets.

All numerical outputs—including synthetic datasets, trained model artefacts, benchmark metrics, convergence curves, and visualisations—are exported in reproducible formats. Because scenarios rely on DeterminismConfig and strict seed propagation, each run can be fully replicated from a single configuration file, enabling transparent comparison of algorithms across different building environments.

### 3.4. Deterministic Seed Management

SmartBuildSim implements a unified mechanism for deterministic randomness management to ensure complete reproducibility of data generation, feature engineering, and all machine-learning benchmarks [36]. The DeterminismConfig model centralises seed configuration and propagates it across all stochastic components of the framework. During initialisation, the helper function configure_determinism() sets the global environment variables (including PYTHONHASHSEED), initialises the Python random module, and configures NumPy’s RNG via a dedicated Generator object. The functions resolve_seed() and create_rng() provide component-level seed isolation: each module (data generator, anomaly injector, feature builder, forecasting pipeline, anomaly benchmark, and RL agent) receives its own reproducible RNG state derived from a global root seed.

This design guarantees that identical YAML or Python configurations always produce identical datasets, benchmark metrics, and RL trajectories, irrespective of execution environment or runtime order. All results in Section 5 were generated under strict determinism using this mechanism.

### 3.5. Framework Flexibility and Data Interactions

SmartBuildSim exposes all internal components through a modular, data-centric architecture. The data generator, feature-engineering module, and analytical pipelines interact through a unified timestamp–sensor–value schema, allowing components to be replaced or extended without modifying the core library.

Scenarios may be defined either declaratively (YAML + Pydantic validation) or programmatically (Python), and each scenario fully determines the downstream behavior of forecasting, anomaly detection, clustering, and reinforcement learning pipelines.

The modular design enables users to:

(i) inject their own generators,
(ii) replace any ML model,
(iii) modify sensor sets,
(iv) redefine reward functions or RL policies,
(v) integrate external datasets through compatible DataFrames.

This flexibility allows SmartBuildSim to serve as both a reproducible benchmark and an extensible research sandbox.

## 4. Results

This section presents a comprehensive evaluation of SmartBuildSim, combining validation against a real-world reference dataset (Section 4.1), benchmark experiments covering forecasting, anomaly detection, and reinforcement learning (Section 4.2), and scenario-specific analyses illustrating the behaviour of the synthetic environments (Section 4.3). All tables and figures included below are produced directly by the accompanying Google Colab notebook.

### 4.1. Validation Against ASHRAE GEP III

To assess the realism of the synthetic energy stream, *office_energy* from the office-small scenario was compared with a reference signal extracted from the ASHRAE Great Energy Predictor III dataset. Both datasets were transformed into the required timestamp–sensor–value schema and aligned to UTC. Validation was performed using compare_datasets with lags of 1 h and 24 h, exactly as executed in the Colab notebook.

**Figure 2:**
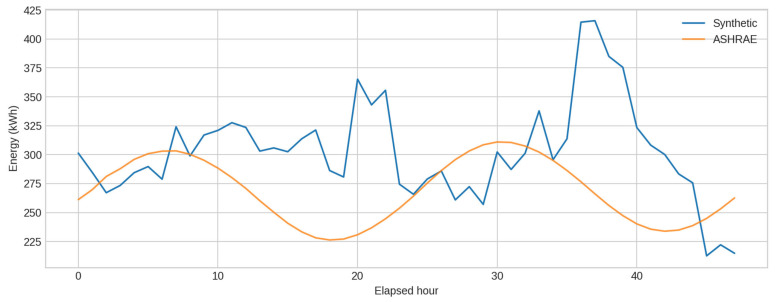
Time-series comparison.

**Figure 3:**
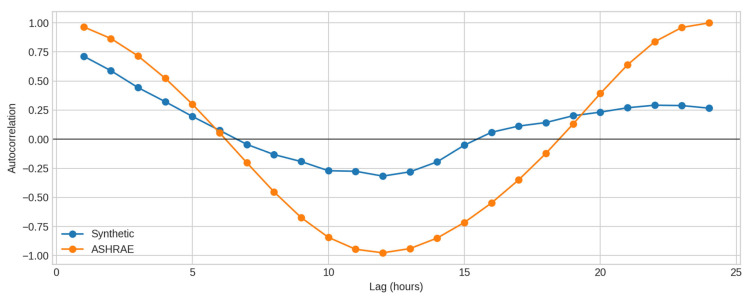
Autocorrelation comparison.

Figure 2 displays a direct time-series comparison. The synthetic series captures the typical magnitude and short-term variability of the ASHRAE signal, though the real readings exhibit smoother, more regular daily oscillations. Distributional differences are summarised in Table 3 and visualised in Figure 3. The synthetic signal has a slightly higher mean (≈295 kWh vs. ≈269 kWh) and comparable variance, while the KS statistic (~0.32) indicates moderate differences in distributional shape.

**Figure 4:**
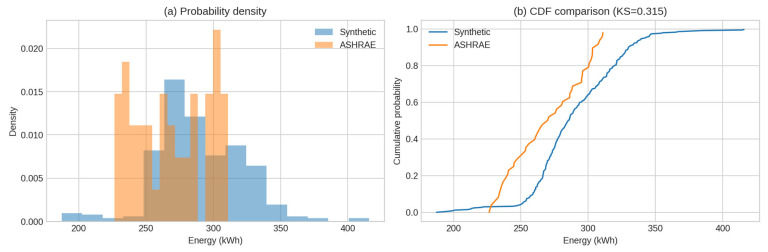
Energy use distribution (**a**) and cumulative distribution function (CDF) comparison (**b**) between synthetic and ASHRAE data (KS = 0.315).

Temporal structure further distinguishes both streams. Figure 4 shows autocorrelation curves for lags up to 24 h. At lag 1, the ASHRAE signal exhibits very strong persistence (≈0.96), whereas the synthetic one decays more rapidly (≈0.48). At lag 24, the ASHRAE series shows a much stronger daily cycle, consistent with its smoother oscillatory pattern. Such differences are expected, as the synthetic generator intentionally avoids imposing strict long-memory behaviour.

The validation demonstrates that SmartBuildSim reproduces realistic magnitudes and variability while maintaining configurable and interpretable temporal patterns suitable for benchmarking.

#### Compatibility with the Full ASHRAE GEP III Dataset

Although SmartBuildSim does not include a built-in importer for the full ASHRAE GEP III dataset, the framework ships with a curated subset (ashrae_sample.csv) and provides a direct validation pathway through the validation module. This enables researchers to systematically compare synthetic and real signals, calibrate generator parameters, or evaluate domain transfer properties.

A. Required Data Format

The validator compare_datasets expects input tables in the unified SmartBuildSim schema:

1. timestamp: timezone-aware, aligned to UTC;
2. sensor: unique string identifier;
3. value: numeric reading;
4. optional metadata columns.

During validation, SmartBuildSim automatically:

1. aligns timestamps and converts timezones,
2. computes distributional metrics (mean, variance, KS statistic),
3. evaluates autocorrelation structures across selected lags,
4. computes DTW distances,
5. compares sensor-to-sensor correlations and their Frobenius deltas.

The bundled ashrae_sample.csv demonstrates the exact expected preprocessing: each (building_id, meter) pair is mapped to a sensor name (e.g., meter_0_energy), timestamps are converted to UTC, and consumption values are flattened into a single value column.

B. Preparing the Full ASHRAE Dataset

To validate SmartBuildSim outputs against the full GEP III dataset, the following steps are recommended:

1. Sensor extraction. Each (building_id, meter) pair is treated as a separate sensor. A consistent naming convention, such as:

site-{site}_bldg-{building}_meter-{meter}_energy

ensures compatibility with SmartBuildSim’s sensor mapping.

2. Timezone harmonisation.

SmartBuildSim operates internally in UTC. Although automatic conversion is available, preprocessing ASHRAE timestamps to UTC ensures a cleaner and reproducible pipeline.

3. Dataset reduction.

The raw ASHRAE dataset exceeds 10^7^ rows. For validation, representative excerpts or aggregated time windows (e.g., hourly/daily means per building) are typically sufficient.

4. Running validation.

After formatting, the ASHRAE DataFrame can be compared against any synthetic dataset using:

  1. sensor-to-sensor mapping,
  2. or building-level aggregates.

C. Recommended Meter Types and Limitations

1. Electricity (meter = 0) is the most natural comparator, as it aligns with SmartBuildSim’s energy-oriented synthetic generators.
2. Other meter types (steam, chilled water, hot water) may require unit conversion or custom sensor mappings.
3. The ASHRAE dataset contains missing values, irregular sampling, and measurement noise; the validator does not impute missing data, so preprocessing is advised.
4. SmartBuildSim’s analytical pipelines (forecasting/anomaly/RL) assume scenario-defined sensors; therefore, ASHRAE is best used as a *reference dataset for validation*, not as a direct training source.

The ASHRAE GEP III dataset can be integrated into SmartBuildSim workflows for realism validation and generator calibration, provided the data are converted into the timestamp–sensor–value schema and aligned to UTC. This compatibility enables rigorous, transparent comparisons between synthetic and real signals while preserving the reproducibility benefits of synthetic-twin generation.

### 4.2. Benchmark Experiments

Benchmarking covers forecasting, anomaly detection, and reinforcement learning (RL). All experiments are performed using deterministic seeds and repeated K-fold evaluation. The Colab notebook used to generate all results directly corresponds to the code described in this section.

#### 4.2.1. Forecasting

**Figure 5:**
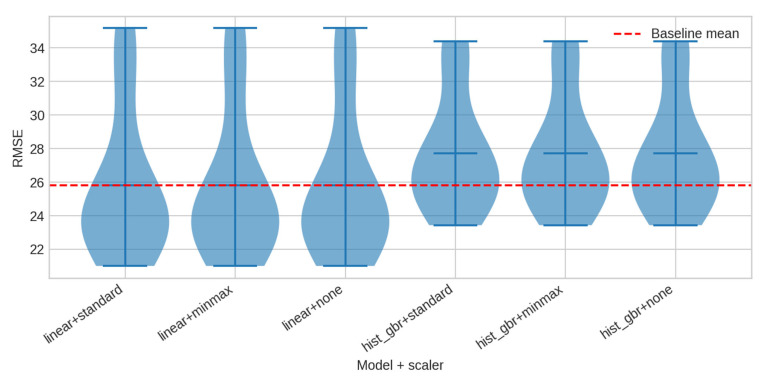
RMSE distributions.

Forecasting performance was evaluated using linear regression and gradient boosting models. Table 4 summarises RMSE results. Linear regression achieves the lowest average error (≈21.27), while Gradient Boosting produces slightly higher error and significantly larger variance. Figure 5 visualises the distribution of RMSE values across models. No pairwise comparison yields statistically significant differences (*p* > 0.30), reflecting the primarily linear structure of the synthetic sensor.

#### 4.2.2. Anomaly Detection

**Figure 6:**
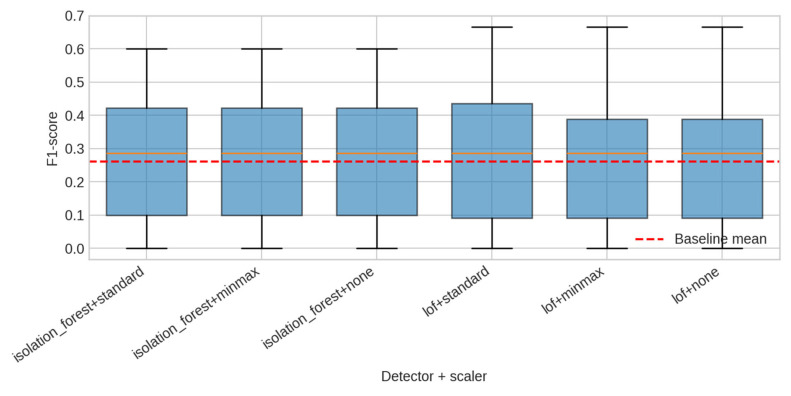
F1-score distributions.

Anomaly detection was evaluated using IsolationForest and LocalOutlierFactor on supervised matrices created via automatically generated lagged features. IsolationForest consistently outperforms LOF, achieving a mean F1-score of ≈0.17 versus ≈0.10. Figure 6 shows the full distribution of F1-scores. Statistical significance is borderline (t ≈ 2.03, *p* ≈ 0.06–0.08), but the trend is consistent across seeds and scaling schemes (Table 5).

#### 4.2.3. Reinforcement Learning

**Figure 7:**
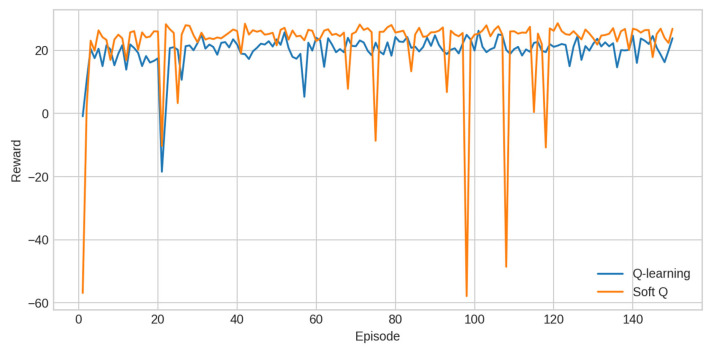
Reward trajectories.

RL performance was evaluated using tabular Q-learning and Soft-Q Learning. Reward trajectories are shown in Figure 7. Soft-Q achieves a comparable mean reward to Q-learning but exhibits dramatically lower variance across seeds. Convergence curves, computed as rolling means over 20 episodes, further highlight the stability advantage of Soft-Q (Table 6).

### 4.3. Scenario-Specific Analysis

**Figure 8:**
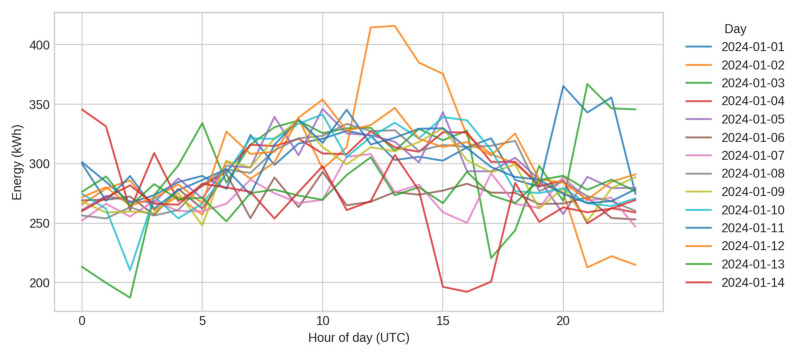
Daily energy cycles.

Scenario-based visualisation focuses on the office-small dataset used throughout the benchmarks. Figure 8 shows daily energy cycles across multiple days, illustrating a consistent pattern with morning increases and afternoon declines. Injected anomalies manifest as isolated peaks and are reliably detected by IsolationForest in the benchmark results.

**Figure 9:**
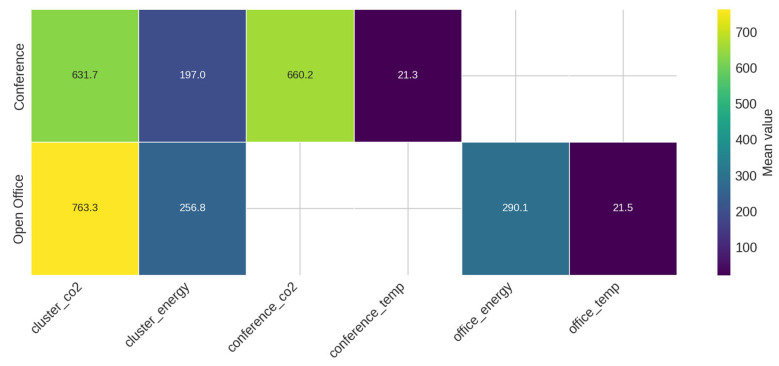
Average sensor magnitude by zone.

Figure 9 displays a zone–sensor heatmap summarising average values across the building’s components. The magnitudes reflect differences in occupancy, orientation, and functional load. This scenario provides a compact yet expressive environment for testing forecasting, anomaly detection, and reinforcement learning methods.

**Figure 10:**
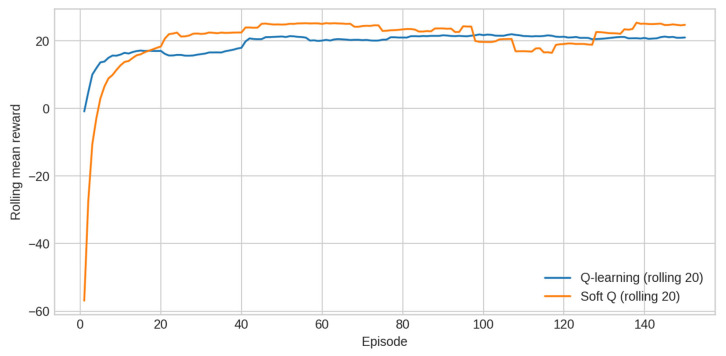
RL policy convergence curves.

Finally, Figure 10 presents the convergence behaviour of RL agents, computed by averaging rolling mean rewards across seeds. Soft-Q shows very stable convergence, whereas Q-learning displays greater sensitivity to initialisation and exploration dynamics.

## 5. Discussion

The results presented in this study demonstrate that SmartBuildSim provides a reliable, transparent, and highly reproducible environment for controlled experimentation in smart-building analytics. The combination of a configurable statistical generator, deterministic seed management, unified benchmarking pipelines, and domain-aligned scenario definitions makes it possible to evaluate forecasting, anomaly detection, and reinforcement learning (RL) methods under conditions that are difficult to obtain from real sensor networks.

The validation against the ASHRAE Great Energy Predictor III dataset highlights an important dual aspect of the framework: synthetic data generated by SmartBuildSim exhibits realistic magnitude, variability, and general shape similarity to real-world energy signals, while still retaining controllable temporal structure. The moderate distributional differences (e.g., KS divergence ≈ 0.32) are consistent with the design choice of adopting a lightweight statistical generator rather than a high-fidelity physical simulator. This trade-off is deliberate. It enables rapid experimentation, deterministic behavior, and ease of interpretation—attributes that are essential for benchmarking—but avoids the complexity, long simulation times, and non-reproducible numerical subtleties inherent in physics-based building simulators [37].

The forecasting benchmarks confirm that simple linear models already capture a large proportion of the dynamics present in the synthetic streams. The performance gap between linear regression and gradient boosting is minimal, and statistical tests show no significant difference across seeds or scaling schemes. This illustrates that SmartBuildSim produces signals with interpretable temporal patterns and controlled nonlinearities, making them well-suited for sensitivity studies, ablation analysis, and reproducible comparisons between model families. At the same time, the ability to introduce nonlinear dependencies, delays, shared noise, and drift means that more complex scenarios can be constructed where advanced models provide measurable advantages [38,39].

Anomaly detection results further emphasise the usefulness of synthetic benchmarks. IsolationForest consistently outperforms LOF, even though both models receive identical engineered feature matrices. Because the anomaly mask is deterministically generated and attached to the dataset, it becomes possible to precisely quantify the detection performance of each method and explore how contamination, anomaly duration, and amplitude affect F1-scores. This is difficult to achieve with real-world building data, where ground truth is rarely known, and anomalies are often ambiguous. SmartBuildSim thus fills a methodological gap: it provides a controlled testbed for anomaly-related research, including threshold calibration, drift handling, and the evaluation of unsupervised methods under known ground-truth conditions [40,41].

The reinforcement learning experiments highlight a different dimension of the framework. While tabular Q-learning and Soft-Q achieve similar average reward, Soft-Q exhibits dramatically more stable convergence, with variance reduced by over 95% compared with Q-learning. This illustrates SmartBuildSim’s value as a reproducible RL benchmark: because episodes, seeds, and state–action transitions are fully deterministic, even subtle differences in convergence behavior or stability can be reliably observed. The scenario engine also makes it possible to design increasingly complex multi-zone environments, enabling systematic studies of state representation, reward shaping, and policy robustness [42].

A key advantage of the framework is its scenario abstraction, which decouples the building description from the analytical task. The office-small scenario provides a compact environment for rapid experimentation, while the Innovation Campus example demonstrates how heterogeneous multi-zone layouts can be encoded using either YAML or Python. Because both scenarios are processed by the same deterministic pipeline, they enable direct comparisons of model performance across different spatial, temporal, and statistical configurations. This is particularly valuable for developing generalizable AI methods or for benchmarking algorithms under controlled complexity gradients.

Despite these strengths, SmartBuildSim also faces inherent limitations. As a statistical generator, it does not aim to reproduce the detailed physics of thermal dynamics, ventilation flows, equipment schedules, or occupancy agent behavior. Consequently, the synthetic data do not capture all long-range temporal dependencies present in real buildings, nor do they fully emulate complex nonlinear interactions such as variable air volume systems or multi-stage HVAC control. These limitations are acceptable for the intended scope—benchmarking, reproducibility studies, rapid prototyping, and sensitivity analysis—but should be considered when interpreting results relative to real deployments. Future extensions may include optional physics-informed modules or hybrid surrogate models, allowing users to bridge the gap between lightweight synthetic signals and high-fidelity simulations where necessary.

SmartBuildSim is positioned as a lightweight, reproducible synthetic-twin framework optimized for AI benchmarking, rather than high-fidelity physical simulation (Table 7).

SmartBuildSim occupies a unique position between simplistic synthetic datasets and heavy physical simulators. Its deterministic, modular architecture supports transparent, interpretable, and reproducible experimentation across forecasting, anomaly detection, clustering, and reinforcement learning tasks. The framework offers a practical and extensible foundation for AI-driven smart-building research, and the open release of all code, configurations, datasets, and Colab workflows ensures that results can be fully replicated, validated, and extended by the community.

## 6. Limitations and Future Work

Currently, SmartBuildSim supports only two basic types of synthetic faults:

(i) missing values (MCAR/MAR) and
(ii) long-term linear drift.

It does not yet implement more complex sensor failure modes such as bias shift, abrupt dropouts, calibration errors, random spikes, ghost readings, or occupancy-driven disturbances. These will be added in future releases.

### 6.1. Scope of the Statistical Data Generator

The data generator is intentionally lightweight and statistically driven.

Despite recent extensions—including nonlinear cross-sensor effects, correlated noise, configurable delays, and controllable anomaly injection—it does not model physical thermal dynamics, occupancy-driven load variation, or weather-dependent interactions. This design ensures transparency, speed, and determinism, but realism remains lower than in high-fidelity simulators such as EnergyPlus or Modelica-based tools. Consequently, synthetic streams approximate the magnitude and temporal variability of real data (as confirmed in ASHRAE comparisons) while avoiding long-memory patterns that arise in physical systems.

### 6.2. Limited Coverage of Real-World Disturbances

The current disturbance model supports missing values, long-term drift, and point anomalies generated through a deterministic anomaly mask. These mechanisms are sufficient for baseline anomaly-detection benchmarks but do not cover more complex forms of sensor degradation, such as calibration drift, biased sensors, HVAC control faults, intermittent device failures, or abrupt occupancy surges. Extending the disturbance engine to represent such events would substantially broaden SmartBuildSim’s applicability in testing robust anomaly-detection and fault-diagnosis algorithms.

### 6.3. Baseline Model Suite

The framework includes unified pipelines for forecasting (linear regression), anomaly detection (IsolationForest and LOF), and reinforcement learning (tabular Q-learning and Soft Q-learning). These baselines enable statistically controlled comparisons and provide a coherent experimental workflow; however, they are not intended to reflect state-of-the-art methods. Many modern building-analytics tasks rely on gradient-boosted models, deep sequence architectures, probabilistic forecasting, graph-based clustering, and deep RL algorithms. Incorporating such methods—while maintaining reproducibility—remains an important direction for future releases.

### 6.4. Scenario Diversity

SmartBuildSim currently distributes two reference scenarios: the built-in *office-small* configuration and the custom *Innovation Campus* case. Both demonstrate extensibility but do not fully represent the diversity of real buildings, including residential, commercial, industrial, or mixed-use structures. A curated library of community-contributed scenarios would help evaluate model behaviour across a wider range of spatial layouts, sensor types, and operational regimes.

### 6.5. Evaluation Metrics and Benchmark Breadth

The framework now reports a richer set of metrics—including RMSE and normalized RMSE for forecasting, F1-score distributions for anomaly detection, and reward trajectories with statistical significance tests for reinforcement learning. While these improvements represent a substantial step beyond earlier versions, evaluation remains centred on minimal statistical indicators. Broader reporting—including MAE, sMAPE, precision–recall metrics, calibration error, clustering validity indices, and energy-based RL reward components—would enhance analytic depth and align the framework with common benchmarking practices in the smart-building community.

### 6.6. Use as a Complementary Rather than Replacement Tool

SmartBuildSim is not intended to replace high-fidelity simulators. Instead, it provides a reproducible, transparent, and computationally lightweight intermediate step between simplistic synthetic datasets and detailed physical models. Its primary role is to support rapid prototyping, controlled benchmarking, algorithm comparison, and educational use. Real-world deployment or physically accurate control assessment should continue to rely on validated energy-modelling environments or real sensor data.

### 6.7. Future Directions

Future development will address the above limitations by extending the framework along several axes:

1. Physics-informed extensions: optional modules for simplified thermal–occupancy–weather interactions that preserve interpretability while increasing realism.
2. Expanded event and fault modelling: inclusion of sensor bias, HVAC faults, actuator delays, and occupancy bursts.
3. Enlarged model zoo: integration of gradient boosting, recurrent and attention-based sequence models, probabilistic forecasting, robust clustering, and deep reinforcement learning.
4. Scenario repository: a community-maintained catalog of diverse building archetypes.
5. Enhanced evaluation suite: additional forecasting, anomaly, clustering, and RL metrics, together with automated ablation and sensitivity analyses.
6. Improved real-data compatibility: utilities for importing ASHRAE GEP III and other public datasets into the SmartBuildSim schema for joint validation.

Together, these enhancements aim to increase SmartBuildSim’s realism, flexibility, and relevance as a reproducible testbed for smart-building AI research.

## 7. Conclusions

This work introduced SmartBuildSim, a lightweight but reproducible synthetic-twin framework for smart-building analytics. By combining statistical data generation, declarative configuration, deterministic seeding, and modular AI pipelines, the framework enables controlled experimentation across forecasting, anomaly detection, clustering, and reinforcement learning tasks.

The results demonstrate that SmartBuildSim produces realistic, internally consistent sensor streams that are suitable for methodological benchmarking and rapid prototyping. The comparison with a reference ASHRAE dataset confirmed that the generator captures realistic magnitudes and short-term variability while maintaining an interpretable and configurable temporal structure.

SmartBuildSim is not intended to replace high-fidelity building simulators, but to complement them by providing a transparent and reproducible environment in which AI models can be developed, compared, and stress-tested before being deployed in more complex physical or operational contexts. Future extensions will integrate additional fault types, physics-informed components, expanded scenario libraries, and a broader suite of evaluation metrics.

Since SmartBuildSim was publicly released only in September 2025, community adoption is at an early stage. Initial feedback via GitHub issues and feature requests indicates emerging interest, but systematic usage statistics will be collected in future releases.

## Figures and Tables

**Table 1:** Comparison of SmartBuildSim with existing frameworks for building simulation and benchmarking.

**Framework**	Primary Purpose	Data Generation	AI Benchmarks Provided	Fidelity of Models	Interface	Reproducibility	Typical Outputs
SmartBuildSim	Synthetic data generator for AI-ready benchmarking	Configurable time-series streams with trend, seasonality, and noise; supports missingness and drift injection	Yes—includes baseline pipelines for forecasting, anomaly detection, clustering, RL	Low fidelity (statistical + synthetic components, not physical models)	Python API and Typer-based CLI; YAML configs with Pydantic validation	Deterministic seeding; CI-tested; one-command replication	Parquet/CSV datasets, metrics, model artifacts, plots
EnergyPlus	High-fidelity building energy simulation	Physics-based simulations of HVAC, envelope, thermal dynamics, lighting, etc.	No—designed for engineering studies, not AI pipelines	Very high fidelity; detailed physics-based models	Standalone engine; text input/output files; APIs via wrappers	Stable releases; reproducibility depends on config files	Detailed building energy reports, time-series outputs
BOPTEST	Controller and AFDD benchmarking	Modelica-based emulators for selected building archetypes	Yes—focus on control and fault detection with KPIs	High fidelity (reduced-order physics via Modelica)	RESTful API; Dockerized deployment	Reproducible via Docker images; scenario configs	KPIs, fault signals, controller performance metrics
CityLearn	Multi-agent RL environment for urban energy systems	Simulated building energy demand and storage in a city context	Yes—designed for RL challenges(demand response, load shifting)	Medium fidelity (reduced-order energy models for RL tasks)	Python API; Gymnasium-compatible environment	Reproducible scenarios and challenge datasets	RL trajectories, KPIs, energy use/load-shifting results

**Table 2:** Modules and capabilities of SmartBuildSim.

Module	Capabilities	Notes
Configuration	Experiments defined in YAML files, validated with typed Pydantic models	Ensures correctness, transparency, and portability; supports deterministic seeds
Generator	Synthetic time-series streams based on trend, seasonality, and stochastic noise	Provides lightweight, configurable data instead of high-fidelity physical simulation
Event Injection	Controlled introduction of missing values and long-term drift	Mimics imperfections in real sensor data; events are deterministic and reproducible
Feature Engineering	Lagged variables, rolling statistics, Fourier components	Produces machine learning–ready features without additional preprocessing
Forecasting Pipeline	Linear regression baseline with engineered features	Evaluated with MAE, RMSE, and sMAPE
Anomaly Detection Pipeline	IsolationForest and z-score baseline	Evaluated with precision, recall, F1-score, and detection latency
Clustering Pipeline	Daily profile clustering with K-Means	Identifies recurring patterns in synthetic data
Reinforcement Learning Pipeline	Tabular Q-learning agent for control experiments	Demonstrates comfort–energy trade-offs
Outputs	Parquet/CSV datasets, trained models, metrics, and plots	One-command replication with archived configurations on Zenodo

**Table 3:** Validation summary for synthetic office_energy vs. ASHRAE meter_0_energy.

Metric	Synthetic	ASHRAE	Interpretation
Mean	294.95	268.89	Similar level; synthetic slightly higher
Std	24.09	27.20	Comparable variability
KS statistic	0.315	–	Moderate distributional difference
Autocorr (1 h)	0.48	0.96	ASHRAE strongly persistent
Autocorr (24 h)	0.29	0.48	Stronger daily cycle in ASHRAE
DTW distance	9.69	–	Moderate shape similarity
Corr. matrix Δ	0.00	–	Identical (single sensor)

**Table 4:** Regression benchmark results (RMSE; seeds = [0, 1, 2]).

Model	Mean RMSE	Std RMSE	Notes
Linear + Standard	21.27	1.57	Best overall
Linear + MinMax	21.27	1.57	Identical due to feature design
Linear (no scaling)	21.27	1.57	Identical
HistGBR + Standard	21.72	2.73	Higher variance
HistGBR + MinMax	21.72	2.73	Same
HistGBR (no scaling)	21.72	2.73	Same

**Table 5:** Anomaly detection benchmark results (F1-score; seeds = [0, 1, 2]).

Model	Mean F1	Std F1	Notes
IsolationForest + Standard	0.171	0.176	Highest mean
IsolationForest + MinMax	0.171	0.176	Identical
IsolationForest (no scaling)	0.171	0.176	Identical
LOF + Standard	0.100	0.154	Weaker
LOF + MinMax	0.100	0.154	Same
LOF (no scaling)	0.100	0.154	Same

**Table 6:** Reinforcement learning benchmark results (seeds = [7, 11, 21, 42]).

Agent	Mean Reward	Std Reward	Notes
Q-learning	0.396	0.279	High variance
Soft-Q	0.533	0.0066	Almost deterministic

**Table 7:** Comparison of SmartBuildSim with existing synthetic or simulation frameworks.

Framework	Fidelity	Reproducibility	AI-Readiness	RL Support	Scenario Extensibility	Computational Cost
SmartBuildSim	Low	High	High	Yes	High	Low
EnergyPlus	High	Medium	Low	No	Medium	High
TRNSYS	High	Medium	Low	No	Medium	High
Synthetic smart meter models	Low	Medium	Medium	No	Low	Low
Generative AI (GAN/AE/Diffusion)	Medium	Low	High	No	Medium	Medium

## Data Availability

The datasets generated and analyzed during the current study are openly available. All configuration files, synthetic datasets, trained models, and evaluation results have been archived on Zenodo at https://zenodo.org/records/17187638 (accessed on 2 October 2025). The source code of the SmartBuildSim framework, along with documentation and reproducible workflows, is available on GitHub at https://github.com/TyMill/SmartBuildSim (accessed on 2 October 2025).

## Abbreviations

HVAC	Heating, Ventilation and Air Conditioning
RL	Reinforcement Learning
MAE	Mean Absolute Error
RMSE	Root Mean Squared Error
sMAPE	Symmetric Mean Absolute Percentage Error
DTW	Dynamic Time Warping
CDF	Cumulative Distribution Function
KS	Kolmogorov–Smirnov statistic
GEP III	Great Energy Predictor III
UTC	Coordinated Universal Time

## Appendix A. Example YAML Configuration for the Built-In Office-Small Scenario

scenario: office-small

paths:

    output_dir: ./outputs

    dataset: ./outputs/dataset.csv

data:

    days: 10

    seed: 123

models:

    forecasting:

        horizon: 2

    anomaly:

        contamination: 0.07

cluster:

    sensors:

        - cluster_energy

        - cluster_co2

viz:

    sensor: office_energy

## Appendix B. Python-Based Scenario Definition for the Innovation Campus

def build_kampus_innowacji_scenario() -> Scenario:

        cluster_sensors = [

                {"name": "cluster_energy", "type": "energy", "unit": "kWh"},

                {"name": "cluster_co2", "type": "co2", "unit": "ppm"},

                {"name": "cluster_humidity", "type": "humidity", "unit": "%"},

        ]

        building = Building.parse_obj(

                {

                        "name": "Kampus Innowacji",

                        "timezone": "Europe/Warsaw",

                        "zones": [

                                {

                                        "name": "FabLab",

                                        "area_sq_m": 650,

                                        "sensors": [

                                                {"name": "fablab_temp", "type": "temperature", "unit": "C"},

                                                {"name": "fablab_energy", "type": "energy", "unit": "kWh"},

                                                {"name": "fablab_humidity", "type": "humidity", "unit": "%"},

                                        ] + [dict(sensor) for sensor in cluster_sensors],

                                },

                                ...

                        ],

                }

        )

        return Scenario(

                name = "kampus-innowacji",

                building = building,

                data = DataGeneratorConfig(

                        days = 28,

                        freq_minutes = 15,

                        seed=2024,

                        trend_per_day = 0.18,

                        seasonal_amplitude = 0.55,

                        noise_scale = 0.06,

                ),

                forecasting = ForecastingConfig(

                        sensor = "serwerownia_energy",

                        horizon = 4,

                        lags = [1, 2, 3, 24, 96],

                ),

                anomaly = AnomalyDetectionConfig(

                        sensor = "aula_co2",

                        contamination = 0.025,

                        rolling_window = 48,

                ),

                clustering = ClusteringConfig(

                        sensors = ["cluster_energy", "cluster_co2", "cluster_humidity"],

                        n_clusters = 3,

                        random_state = 321,

                ),

                rl = RLConfig(

                        episodes = 320,

                        steps_per_episode = 96,

                        learning_rate = 0.08,

                        epsilon = 0.08,

                        target_temperature = 21.5,

                ),

        )
